# SULF2 promotes tumorigenesis and inhibits apoptosis of cervical cancer cells through the ERK/AKT signaling pathway

**DOI:** 10.1590/1414-431X20198901

**Published:** 2020-02-07

**Authors:** Tao Jiang, Zhao-Hui Chen, Zhe Chen, Dan Tan

**Affiliations:** 1 Department of Obstetrics and Gynecology, the First People's Hospital of Jingzhou City, Jingzhou, Hubei Province, China; 2 Department of Women's Tumor, Jingzhou Cancer Hospital, Jingzhou, Hubei Province, China; 3 Department of Obstetrics and Gynecology, the Second People's Hospital of Jingzhou City, Jingzhou, Hubei Province, China

**Keywords:** SULF2, Cervical cancer, ERK/AKT, Proliferation, Apoptosis

## Abstract

The objective of this study was to explore the role of the SULF2-mediated ERK/AKT signaling pathway in cervical cancer. SULF2 expression was detected in tumor tissues and tumor-adjacent normal tissues from cervical cancer patients. HeLa cells were divided into six groups: control group, NC group, SULF2 siRNA group, SULF2 group, SULF2 + LY294002 group, and SULF2 + U0125 group. In each group, HeLa cells received the corresponding treatment, followed by measurement of the cellular biological characteristics and expression of the ERK/AKT signaling pathway. We also confirmed the effect of SULF2 *in vivo* using a xenograft model in nude mice. SULF2 was upregulated in cervical cancer tissues, which was specifically associated with the clinical stage, histological differentiation, and lymphatic metastasis. Compared to the control group, the SULF2 siRNA group displayed decreased expression of SULF2, concomitant with reduced proliferation, migration, and invasion, but there was an increase in the apoptosis rate of HeLa cells, as well as downregulation of the p-Akt/Akt, p-ERK/ERK, and Bax/Bcl-2 ratios and cyclin D1. Additionally, tumor growth was significantly inhibited in the xenograft model of nude mice. The results in the SULF2 group were quite the opposite in which SULF2 facilitated the growth of cervical cancer cells, which was reversed by LY294002 or U0126. SULF2 is highly expressed in cervical cancer, and thus, downregulation of SULF2 can inhibit the ERK1/2 and AKT signaling pathways to suppress the proliferation, invasion, and migration of cervical cancer cells while facilitating apoptosis.

## Introduction

Cervical cancer is currently the second leading cause of cancer-related death in females worldwide, second to breast cancer (1). According to global estimates, approximately 500,000 new cases of cervical cancer are diagnosed, 80% of which originate from developing countries, and 233,000 people die of this type of cancer worldwide every year (2). Although current treatments have resulted in great advancements in controlling tumor growth, most patients in the mid-term or advanced stage are still suffering from poor efficacy and prognosis (3,4). As reported previously, cervical cancer is a multistage and multigene network process involving epigenetic variation, immunological changes, and inactivation of tumor suppressor genes or activation of oncogenes (5,6). Thus, investigating the pathogenesis of cervical cancer at the molecular level is of great significance for the treatment of cervical cancer.

The sulfatase family, consisting of sulfatase 1 (SULF1) and sulfatase 2 (SULF2), is involved in multiple physiological and pathological processes by regulating the sulfation status of heparan sulfate proteoglycan, which has become one of the hot spots in cancer research (7–9). The SULF2 gene is located on human chromosome 20q13, which has been reported to play important roles in various cancers, including liver cancer (10), gastric cancer (11), kidney cancer (12), breast cancer (9), and non-small cell lung cancer (13). On the other hand, extracellular signal-regulated kinase is one of the members of the mitogen-activated protein kinases (MAPKs), and once activated, it is a key step in malignant cell transformation and the development and progression of tumors (14,15). Evidence has confirmed the involvement of the ERK1/2 signaling transduction pathway in the development of cervical cancer (16,17). Moreover, AKT, also known as protein kinase B, is a downstream target protein of PI3K and, as the core element of the PI3K/AKT signaling pathway, is crucial in the evolution of tumors, including promoting cell proliferation, anti-apoptosis, and pro-angiogenesis via initiating the downstream substrate (18–20). Studies have shown the close association of the AKT pathway with cervical cancer progression (21,22). More notably, SULF2 was found by Lai et al. (10) to be upregulated in liver cancer, and its overexpression could facilitate the growth and migration of liver cancer cells by enhancing the phosphorylation of ERK and AKT. In addition, Peterson et al. (23) uncovered that the stable expression of SULF2 could inhibit the ERK signaling pathway to suppress the proliferation and invasion of breast cancer cells, which was verified in the xenograft models of nude mice. However, there remains little information regarding the association between SULF2 and cervical cancer, and we postulated that SULF2 may exert its effect in the development of cervical cancer by regulating the ERK/AKT signaling pathway.

Collectively, in this study, we aimed to explore the mechanism of the SULF2-mediated ERK/AKT signaling pathway in cervical cancer to provide theoretical evidence and an experimental foundation for the development of clinical treatments for cervical cancer.

## Material and Methods

### Ethics statement

The study protocol was reviewed and approved by the Ethical Committee of the First People's Hospital of Jingzhou City, and prior to enrollment, all subjects and volunteers were informed of the content of this study and signed written informed consent. Animal experiments were all conducted under the Guide for the Care and Use of Laboratory Animals (24).

### Subjects

A total of 79 tumor tissues and the tumor-adjacent normal tissues were selected from cervical cancer patients admitted to the Department of Gynecology of the First People's Hospital of Jingzhou City between December 2015 and December 2017. All subjects were between 36 and 67 years of age, with an average age of 53.99±9.95 years, including 23 patients with adenocarcinoma and 56 with squamous carcinoma. According to the staging criteria of cervical cancer of the International Federation of Gynecology and Obstetrics, 47 patients were in stage Ib-IIa and 32 in stage IIb-IIIa. In addition, 57 patients had moderate/high differentiation, while 22 patients had poor differentiation, consisting of 34 patients with lymph node metastasis and 45 patients without lymph node metastasis. All tissue samples were confirmed through pathological diagnosis without any intervention or therapy prior to this surgery.

### Immunohistochemistry

Initially, paraffin sections were dewaxed and hydrated regularly. Then, antigen retrieval was performed using a microwave to expose the antigen, and the endogenous peroxidase was inactivated by incubating with 3% H_2_O_2_ in deionized water for 10 min. The sections were boiled at 95°C for 15 min in 0.01 nM citrate buffer (pH=6.0) and cooled for 30 min. Normal goat serum buffer was added dropwise on each section for 20 min of regular incubation, and without any treatment, sections were directly incubated with the primary antibodies overnight at 4°C, followed by incubation with the secondary antibodies. Sections were then incubated with the streptavidin-peroxidase solution and diaminobenzidine sequentially. Following counterstaining with hematoxylin, sections were observed under the microscope (Olympus, Japan).

### Cell culture

The human cervical cancer cell lines HeLa, SiHa, Ca-Ski, C33A, and C-4I and the immortalized human cervical epithelial cell-H8 cell line were purchased from the American Type Culture Collection (ATCC; USA). Cells were thawed completely and centrifuged at 300 *g* for 5 min at 37°C to collect the sediment, which was later resuspended in serum-free RPMI 1640 medium. Following centrifugation at 300 *g* for 5 min at 37°C, RPMI 1640 medium containing 10% fetal bovine serum (FBS) was added dropwise into the sediment for regular culture and passage. Cells were harvested promptly for the experiment, and the culture conditions were set as 5% CO_2_ and 37°C.

### Cell grouping and transfection

HeLa cells were divided into 6 groups: control group (no transfection), NC group (cells transfected with the negative control siRNA), SULF2 siRNA group (cells transfected with SULF2 siRNA), SULF2 group (cells transfected with the SULF2 plasmid), SULF2 + LY294002 group (cells transfected with the SULF2 plasmid followed by treatment with 20 μM LY294002 for 24 h), and SULF2 + U0126 group (cells transfected with the SULF2 plasmid followed by treatment with 25 μM U0126 for 24 h). LY294002 and U0126 were provided by R&D Systems (USA), while SULF2 plasmid, SULF2 siRNA, and NC siRNA were designed and synthesized by Shanghai GeneChem Biotech Co., Ltd. (China) Transfection was performed following the instructions of the Lipofectamine^TM^ 2000 manufacturer (Sigma, USA).

### qRT-PCR

TRIZOL reagent was utilized to extract the total RNA from tissues and cells. Following the measurements of the concentration and purity of RNA using an ultraviolet spectrometer (Ocean Optics Inc., USA), cDNA was prepared by reverse transcription of RNA using the PrimeScript^TM^ RT-PCR Kit (TaKaRa Biotechnology Co., Ltd., China). PCR was carried out with the appropriate volume of cDNA as the template, and the primers designed by Primer 5.0 were as follows: SULF2, forward primer, 5′-CTGAATCCCCACATCGTCCTC-3′, reverse primer, 5′-GTCCACCTTGTCATTGTCTCTCTTGT-3′; GAPDH, forward primer, 5′-ACCACAGTCCATGCCATCAC-3′, reverse primer, 5′-TCCACCACCCTGTTGCTTGTA-3′. Primers were synthesized by Nanjing GenScript Biotech Co., Ltd. (China) using the PCR kit (KR011A1, TIANGEN Biotech Co., Ltd., China), qRT-PCR was conducted according to the instructions, and gene expression was calculated by the formula 2^−△△Ct^ (ΔCt = Ct_target gene_ – Ct_internal reference gene_; ΔΔCt = ΔCt_experiment group_ – ΔCt_control group_).

### Western blotting

Human cervical cancer cells (1×10^9^) were obtained, 0.5 mL PBS (pH=7.4) was added, and samples were homogenized by ultrasound, followed by measurement of the concentration of total protein in the supernatant using the Bradford method. The total volume of proteins in each sample was adjusted to the same concentration by adding normal saline. Proteins (50 μg) of each sample were loaded into the upper gel for sodium dodecyl sulfate-polyacrylamide gel electrophoresis (SDS-PAGE) to separate the proteins. Then, proteins in the gel were transferred onto a polyvinylidene fluoride (PVDF) membrane in a semi-dry transfer box (Bio-Rad, USA). Unoccupied sites on the PVDF membrane were blocked with skimmed milk, and following several washes in PBST, proteins on the PVDF membrane were initially probed by incubation with the rabbit anti-human monoclonal antibodies against SULF2 (ab101057, Abcam, UK), p-ERK1/2 (ab214362, Abcam), ERK1/2 (ab17942, Abcam), p-AKT (ab81283, Abcam), AKT (ab8805, Abcam), cyclin D1 (ab16663, Abcam), Bcl-2 (ab32124, Abcam), Bax (ab53154, Abcam), and GAPDH (ab181602, Abcam) for 1 h at room temperature. After 5 washes in PBST (3 min/wash), immunoblots were further incubated with horseradish peroxidase (HRP)-labeled secondary antibodies (Beijing Zhongshan Golden-Bridge Biotech Co., Ltd., China) for 1 h. The protein expression was quantified relative to that of GAPDH as a loading control.

### MTT

Cervical cancer cells in all transfection groups were inoculated onto a 96-well plate (Corning, USA) at a density of 1×10^3^ cells/well. Then, 20 μL of MTT reagent (5 mg/mL, Sigma-Aldrich, USA) was dropped into each well at 12, 24, 48, 72, and 96 h after inoculation. Following 4 h of incubation at 37°C, cells were subjected to absorbance measurement at 490 nm in a microplate reader (Model 3550, Bio-Rad) in the presence of 150 μL of DMSO (Sigma-Aldrich). This experiment was repeated three times.

### Wound healing

After adjustment of the density, the cells were transferred onto a 6-well plate (2 mL/well, i.e., 5.0×10^5^/well) and three replicates were prepared for different treatments. After confluence, an aseptic tip was used to draw a line vertically and slightly on the surface of the medium without any cells in the line. Then, cells that were loosely adhered were removed. In the wells, DMEM supplemented with 10% serum was added for culture at 37°C under 5% CO_2_ for 48 h. At 0 and 48 h, images of the line area were obtained to observe the changes in the cell quantity in the line. This experiment was conducted in triplicate.

### Transwell assay

Briefly, the upper portion of Transwell chambers (Corning) was coated with 40 μL of Matrigel diluted 1:10 in serum-free DMEM at 37°C, and 30 min later, Matrigel was polymerized into the gel. Cell suspension (5×10^5^/mL, 100 μL) was added into the upper chamber of the Transwell plate, followed by culture in 200 μL of serum-free medium; in the lower chamber, 500 μL of complete medium containing 0.05% FBS was added. Then, the plate was transferred into an incubator (37°C, 5% CO_2_) for culture. At 48 h, the upper chamber was removed and fixed in cold 4% paraformaldehyde for 30 min. Thereafter, cells were stained in 0.1% crystal violet for 1 min, dehydrated in gradient ethanol (80, 95, and 100%), and cleared in xylene. From the basement of the upper chamber, the polycarbonate membrane was removed and transferred onto a slide followed by mounting in neutral balsam. Under the microscope (×400), 6 fields of view were selected randomly for cell counting and the results were averaged for three replicates.

### Flow cytometry

Cells were digested in 0.25% EDTA-free trypsin (PYG0107, Boster, China) and collected in a tube for centrifugation at 450 *g* for 5 min to collect the sediment. In cold PBS, cells were washed three times, and the sediment was collected after centrifugation. According to the instructions of the Annexin-V-FITC cell apoptosis detection kit (K201-100, Biovision, USA), Annexin-V-FITC/PI buffer was prepared by Annexin-V-FITC, PI, and HEPES at a ratio of 1:2:50. The cell suspension was prepared as 1×10^6^ cells in 100 µL buffer, and after incubating for 15 min, 1 mL of HEPES buffer was added. At the wavelengths of 515 nM and 620 nM activated by 488 nM, the fluorescent signals of FITC and PI were detected using a band-pass filter to evaluate cell apoptosis. This experiment was also conducted in triplicate.

### Construction of the xenograft models in nude mice

Twenty-four BALB/c mice (age: 4 weeks; weight: 13.5±0.5 g), provided by the Shanghai SLAC Laboratory Animal Co., Ltd. (China), were divided into the control group, NC group, and SULF2 siRNA group (8 mice each). The suspension of HeLa cells in the transfection groups was mixed well by blowing with a pipette, and 200 μL of cells was extracted by a 1-mL syringe and then injected subcutaneously into the left-side axilla of the nude mice. Mental status, food and water intake, activation, and tumor growth were observed daily, and the weight of the mouse and the length (a) and width (b) of the tumor were recorded weekly. Tumor volume was calculated by the formula: TV=1/2ab^2^. A growth curve was prepared for the xenograft tumors according to the change in tumor volume. At the end of the experiment, mice were sacrificed by cervical dislocation to obtain the xenograft tumor tissues, followed by photographing and weighing, as well as measurement of positive expression of Ki67 by immunohistochemistry.

### Statistical analysis

All data were analyzed using SPSS 21.0 software (SPSS Inc., USA). The chi-squared test was used to compare enumeration data. Measurement data in the form of means±SD were compared by the *t*-test between two groups and by one-way analysis of variance (ANOVA) with Turkey's *post hoc* test among groups. P<0.05 indicated a statistically significant difference.

## Results

### Expression of SULF2 in cervical cancer and cells

**Figure 1.**
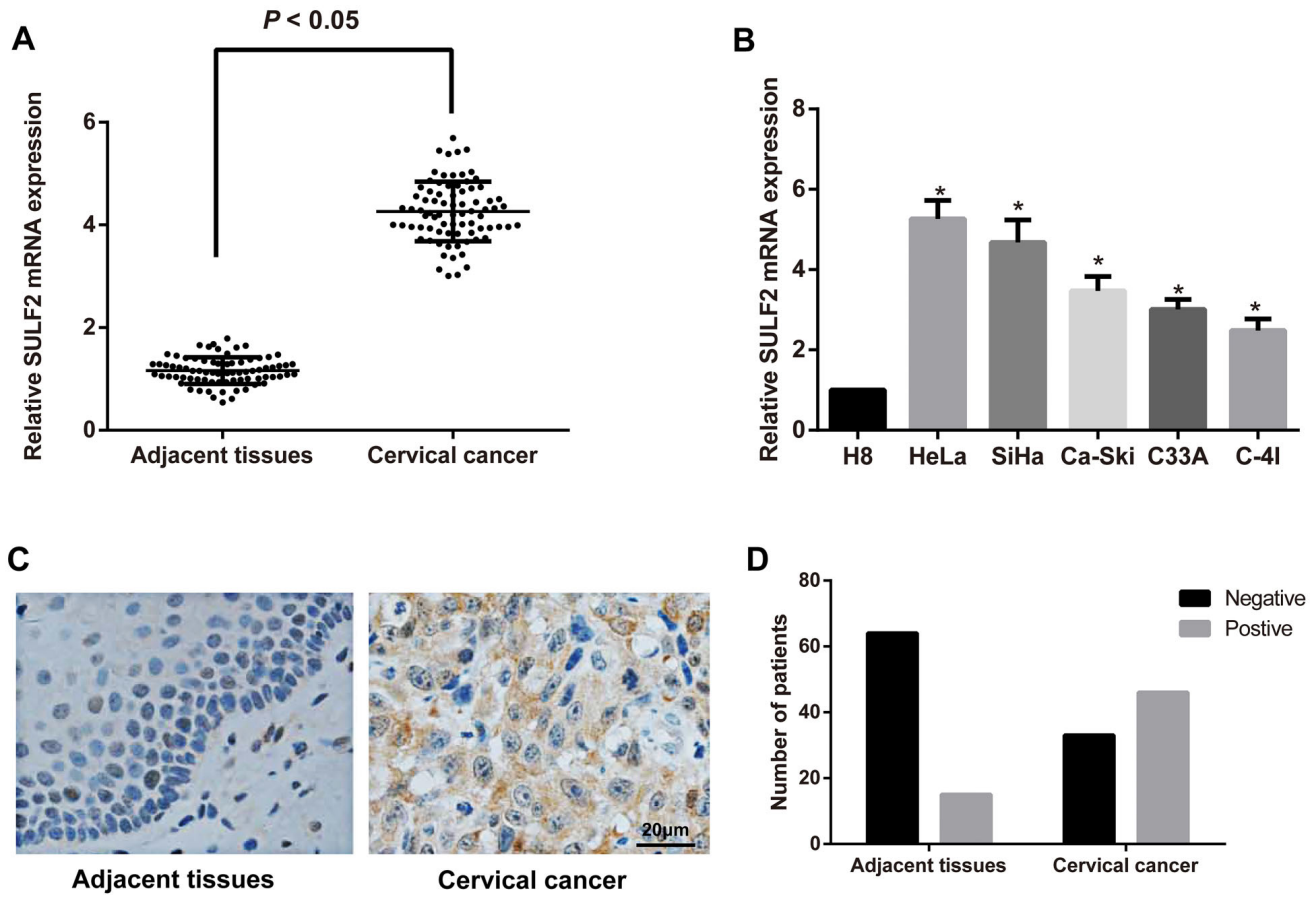
Expression of sulfatase 2 (SULF2) in cervical cancer tissues and cells. **A**, Relative SULF2 mRNA expression in cervical cancer tissues and tumor-adjacent normal tissues measured by qRT-PCR. **B**, Relative SULF2 mRNA expression in the H8, HeLa, SiHa, Ca-Ski, C33A, and C-4I cell lines measured by qRT-PCR. Data are reported as means±SD, *P<0.05 *vs* H8 cells (ANOVA). **C** and **D**, Protein expression of SULF2 in the cervical cancer tissues and tumor-adjacent normal tissues detected by immunohistochemistry (bar: 20 µm).

As shown by qRT-PCR and immunohistochemistry in Figure 1A, C, and D, the mRNA expression of SULF2 was significantly increased in the tumor tissue of cervical cancer (4.26±0.58) compared with that in the matched non-tumor adjacent tissues (1.16±0.26, P<0.05). Among 79 patients with cervical cancer, 46 of 79 (58.23%) cases revealed positive expression of SULF2 relative to their matched non-tumor adjacent tissues (15/79, 18.99%). Statistical analysis (chi-squared test) revealed that SULF2 expression was correlated with the clinical stage (P=0.005), histological differentiation (P<0.001), and lymphatic metastasis (P=0.001). However, SULF2 expression was not associated with other factors, including age (P=1.000), pathological type (P=0.316), and HPV infection (P=0.799) in cervical cancer patients (Table 1). In cell lines, compared to the immortalized cervical epithelial cells (H8 cells), SULF2 mRNA was upregulated significantly in the cervical cancer cell lines HeLa (5.26±0.46), SiHa (4.68±0.56), Ca-Ski (3.47±0.36), C33A (3.01±0.25), and C-4I (2.48±0.29), with the highest expression found in HeLa cells (all P<0.05, Figure 1B). Therefore, the HeLa cell line is an appropriate choice for subsequent experiments.

**Table 1.** Correlation of sulfatase 2 (SULF2) expression with the clinicopathological features of the cervical cancer patients.

Clinicopathological features	N	SULF2 expression		P
		Negative	Positive	
Age				1.000
≥60	34	14	20	
<60	45	19	26	
Pathological type				0.316
Squamous carcinoma	56	21	35	
Adenocarcinoma	23	12	11	
Clinical stage				0.005
Ib-IIa	47	26	21	
IIb-IIIa	32	7	25	
Lymph node metastasis				0.001
With	34	7	27	
Without	45	26	19	
Differentiation				<0.001
Poor differentiation	22	2	20	
Moderate/high differentiation	57	31	26	
HPV				0.799
Positive	58	25	33	
Negative	21	8	13	

Chi-squared test was used for statistical analysis.

### SULF2 expression and cell proliferation in cervical cancer

**Figure 2.**
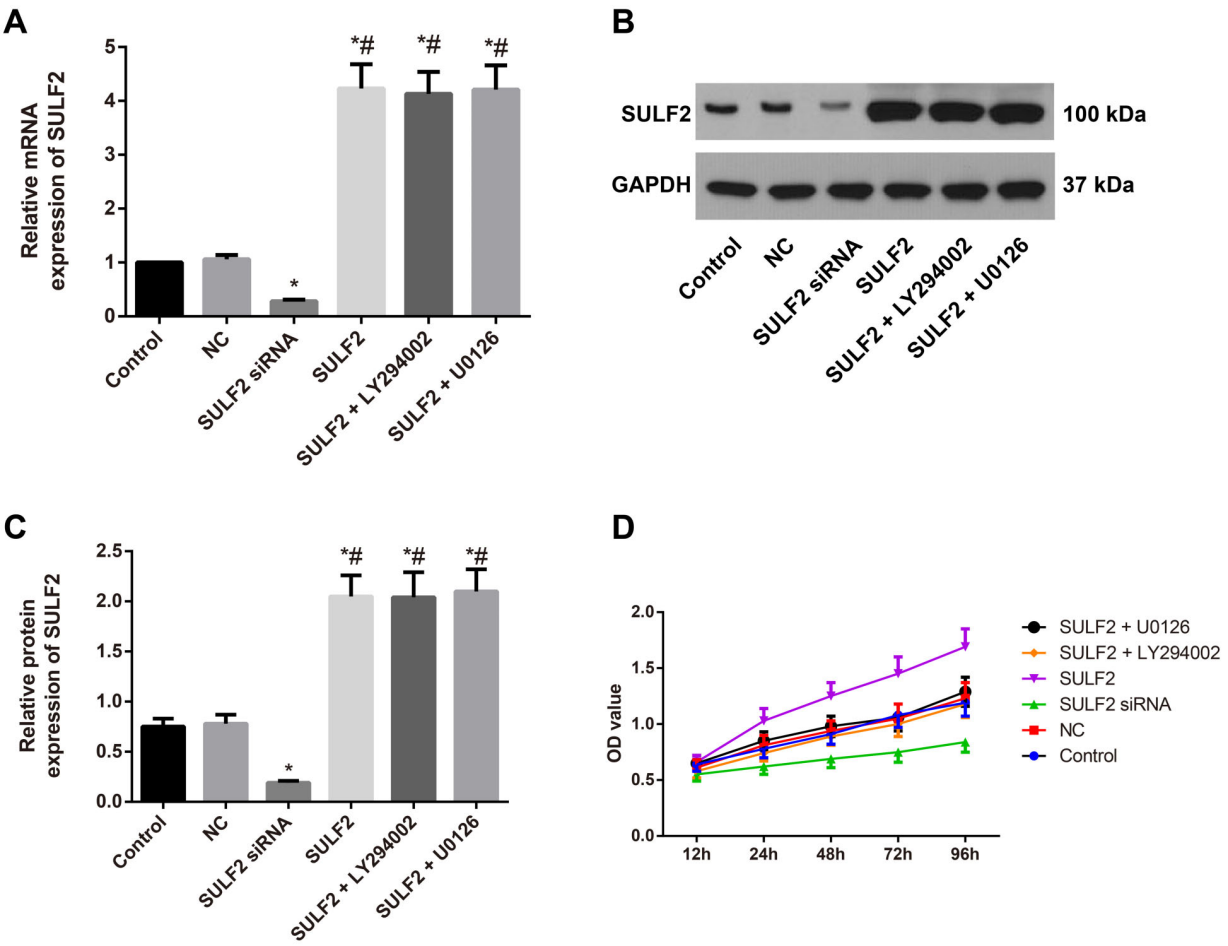
Sulfatase 2 (SULF2) expression and cell proliferation in cervical cancer. **A**, Relative mRNA expression of SULF2 in cervical cancer cells in the transfection groups detected by qRT-PCR. **B** and **C**, Relative protein expression of SULF2 in cervical cancer cells by western blotting. **D**, Cell proliferation of cervical cancer detected by MTT. Data are reported as means±SD. *P<0.05 *vs* the control group; ^#^P<0.05 *vs* the SULF2 siRNA group (ANOVA). NC group: transfected with the negative control siRNA.

First, the mRNA and protein expression levels of SULF2 were found to be significantly downregulated in the SULF2 siRNA group but upregulated in the SULF2 group compared to the control group (all P<0.05). No statistical significance of SULF2 expression was identified between the control group and the NC group (all P>0.05, Figure 2A–C). We found that the cell proliferation of cervical cancer cells with SULF2 knockdown was significantly decreased compared with that of the control cells by MTT assays (P<0.05). In contrast, overexpression of SULF2 significantly promoted cell proliferation compared with control cells (P<0.05). In addition, cells in the SULF2 + LY294002 group and SULF2 + U0126 group had significantly restricted proliferation compared with the SULF2 group (all P<0.05, Figure 2D).

### Invasion and migration of cervical cancer cells affected by SULF2

**Figure 3.**
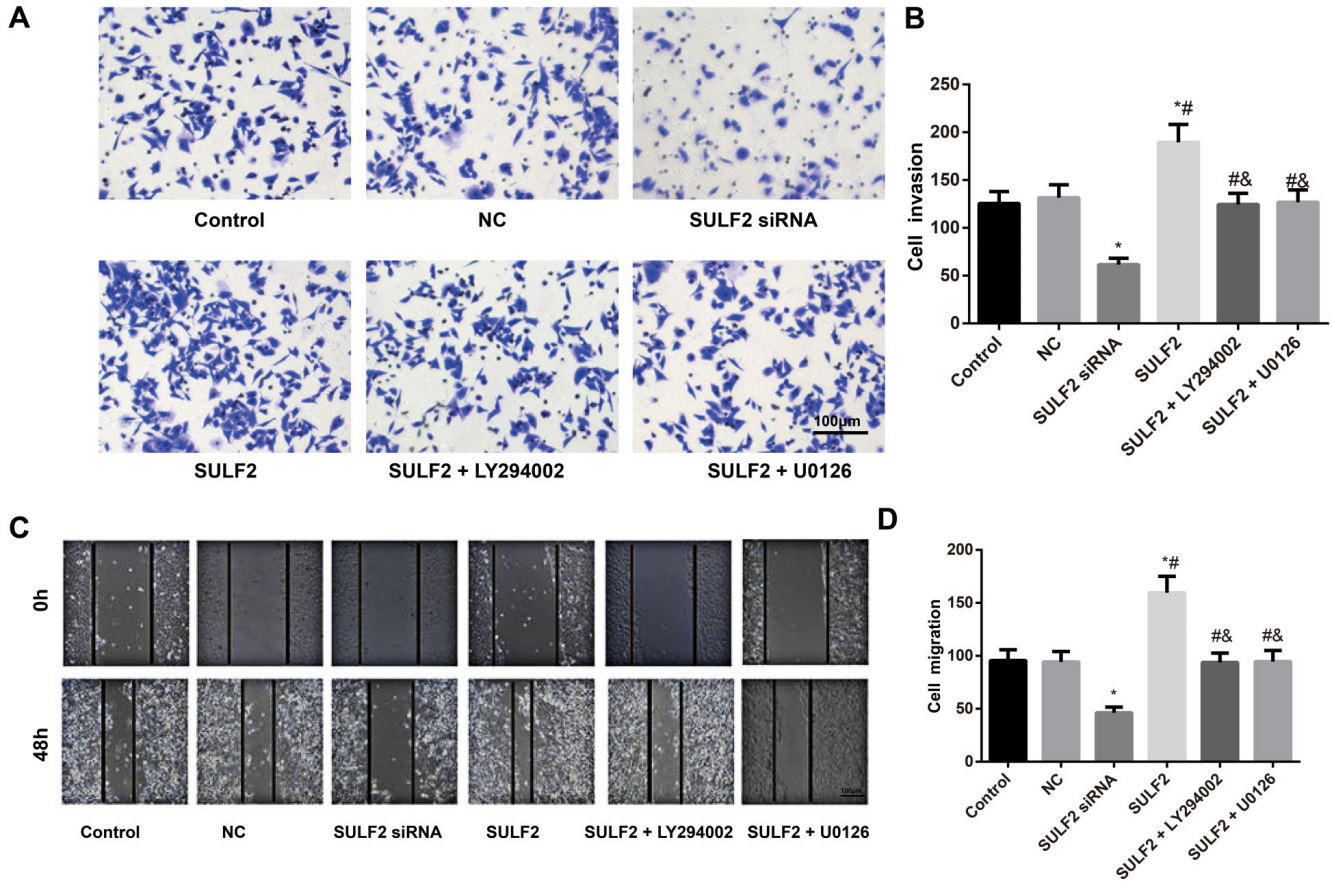
**A** and **B**, Invasive ability of cervical cancer cells detected by the Transwell assay (bar: 100 µm). **C** and **D**, Migration of cervical cancer cells detected by the wound-healing assay. Data are reported as means±SD. *P<0.05 *vs* the control group; ^#^P<0.05 *vs* the sulfatase 2 (SULF2) siRNA group; ^&^P<0.05 *vs* the SULF2 group (ANOVA). NC group: transfected with the negative control siRNA.

The wound-healing assay and Transwell assay both indicated that SULF2 siRNA significantly reduced the number of migrating and invasive cells (all P<0.05, Figure 3). SULF2 overexpression significantly increased the number of migrating and invasive cells (all P<0.05). Moreover, LY294002 and U0126 abolished the promotion of migration and invasion induced by SULF2 overexpression (all P<0.05).

### Apoptosis of cervical cancer cells affected by SULF2

**Figure 4.**
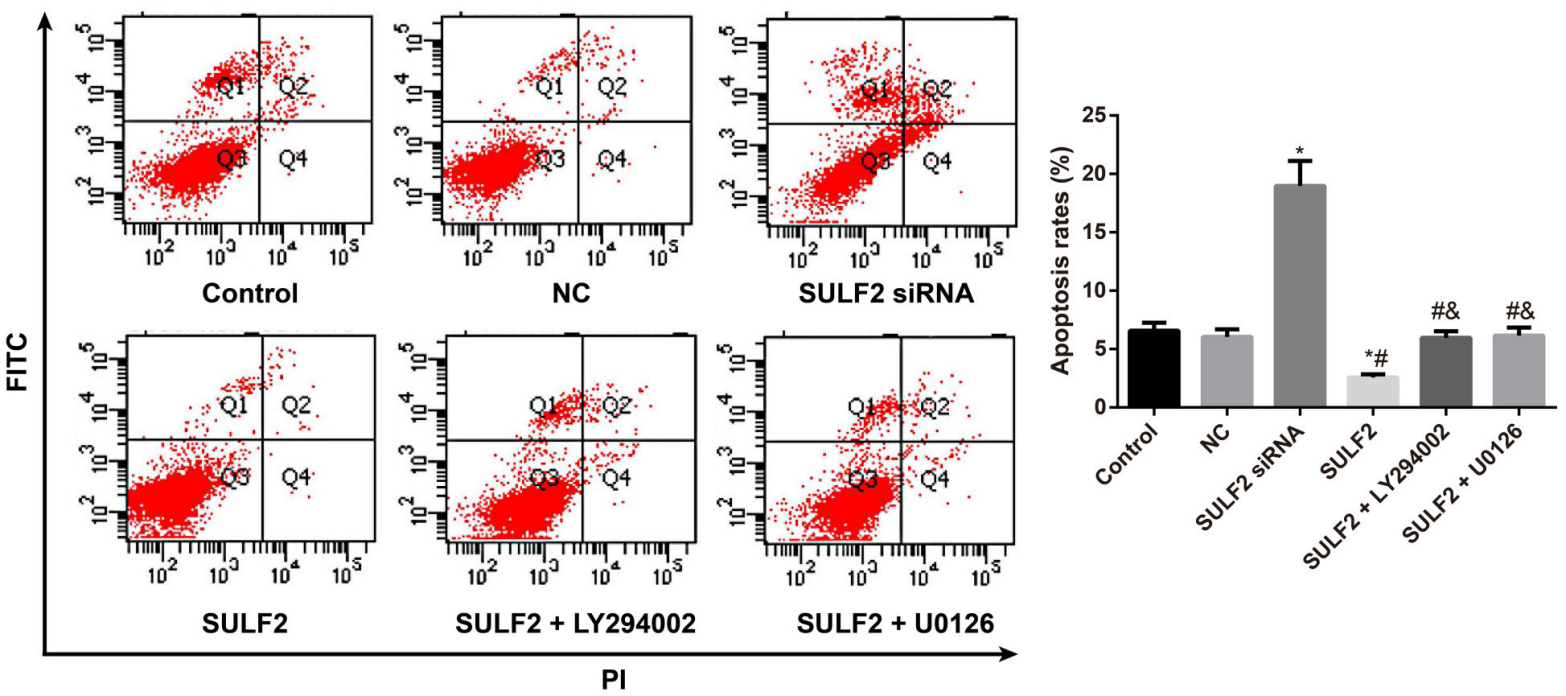
Apoptotic rate of cervical cancer cells in the transfection groups detected by flow cytometry. Data are reported as means±SD. *P<0.05 *vs* the control group; ^#^P<0.05 *vs* the sulfatase 2 (SULF2) siRNA group; ^&^P<0.05 *vs* the SULF2 group (ANOVA). NC group: transfected with the negative control siRNA.

Cell apoptosis was assessed by flow cytometry, and cells were stained with Annexin V-FITC/PI and gated into the lower right (LR) and upper right (UR) quadrants. Cells in the LR and UR quadrants represented early (Annexin V(+)/PI(−)) and late apoptotic (Annexin V(+)/PI(+)) cells, respectively. The apoptosis rates are reported as the sum of the percentages of early and late apoptotic cells. As presented in Figure 4, the apoptosis rate was 18.96±2.15% in the SULF2 siRNA group and 2.58±0.26% in the SULF2 group. The apoptosis rate in the SULF2 siRNA group was enhanced but declined in the SULF2 group (all P<0.05), with no difference between the control group (6.58±0.68%) and NC group (6.06±0.64%) (all P>0.05). In addition, compared to the SULF2 group, administration of LY294002 (5.96±0.58%) or U0126 (6.15±0.69%) led to a significant decrease in the apoptosis rate (all P<0.05).

### ERK/Akt pathway-related protein expression affected by SULF2

**Figure 5.**
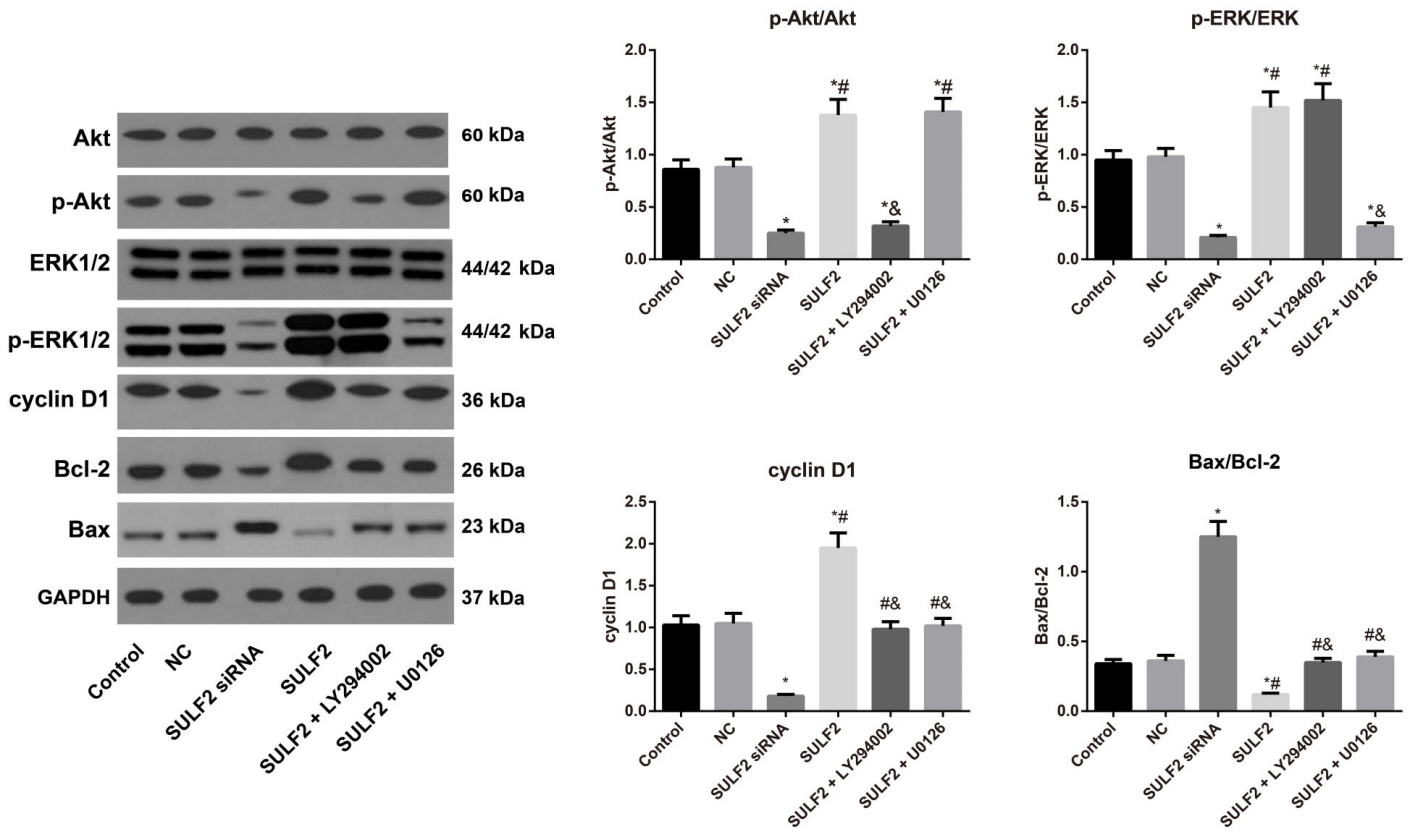
Expression of ERK1/2 and Akt signaling pathways in cervical cancer cells in the transfection groups detected by western blotting assay. Data are reported as means±SD. *P<0.05 *vs* the control group; ^#^P<0.05 *vs* the sulfatase 2 (SULF2) siRNA group; ^&^P<0.05 *vs* the SULF2 group (ANOVA). NC group: transfected with the negative control siRNA.

The western blotting results (Figure 5) demonstrated that the protein expression levels of p-Akt/Akt, p-ERK/ERK, cyclin D1, and Bax/Bcl-2 were significantly lower in the SULF2 siRNA group than those in the control group, whereas the opposite was detected in the SULF2 group (all P<0.05). Furthermore, compared with the SULF2 group, the expression of p-Akt/Akt and Bax/Bcl-2 as well as cyclin D1 was significantly downregulated after LY294002 treatment (all P<0.05), with no significant changes in the p-ERK/ERK ratio (P>0.05). However, treatment with U0126 resulted in decreased p-ERK/ERK, Bax/Bcl-2, and cyclin D1 (all P<0.05), but no statistical significance in the p-Akt/Akt ratio was observed (P>0.05).

### Subcutaneous tumorigenesis of xenografts in nude mice affected by SULF2

**Figure 6.**
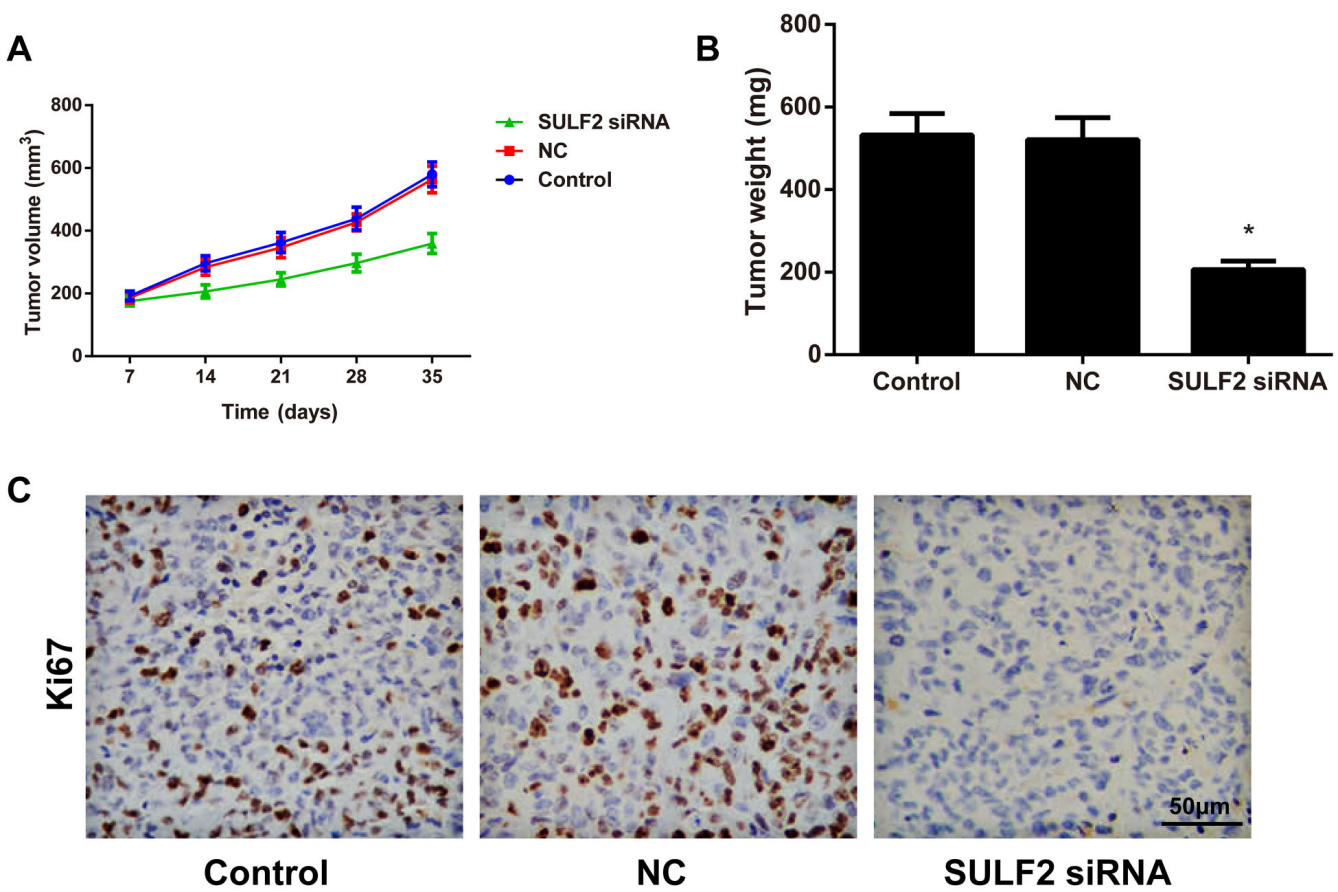
Inhibition effect of sulfatase 2 (SULF2) siRNA on the growth of xenograft tumors in nude mice. **A** and **B**, Comparison of tumor volume and weight of the human cervical cancer xenografts in nude mice. **C**, Positive expression of Ki67 determined by immunohistochemistry (bar: 50 µm). Data are reported as means±SD. *P<0.05 *vs* the control group (ANOVA). NC group: transfected with the negative control siRNA.

As shown in Figure 6A, the tumors formed by the SULF2 siRNA cells grew much slower than did those formed by the control cells (P<0.05). In addition, the weight of the tumors in the SULF2 siRNA group (206.65±20.16 mg) was reduced compared to those in the control group (532.26±51.76 mg) (Figure 6A, P<0.05), while no obvious changes in tumor volume and weight were observed between the control and NC groups (all P>0.05). Moreover, immunohistochemistry analysis confirmed that the positive expression of Ki67 was stained in deep brown. In the SULF2 siRNA group, Ki67 expression was significantly limited, with a lower quantity of slightly stained positive cells than in the control group (Figure 6C, all P<0.05).

## Discussion

SULF2, the extracellular sulfatase that changes the sulfation of heparan sulfate proteoglycans, was significantly upregulated in cervical cancer, and its higher expression was significantly associated with the clinical stage, histological differentiation, and lymphatic metastasis of cervical cancer, which was consistent with other studies. Evidence from an investigation on liver cancer suggested that overexpressed SULF2 predicted a worse prognosis (25). In addition, SULF2 expression might be credited as a valuable diagnostic and prognostic biomarker in non-small cell lung cancer (26). Furthermore, Flowers et al. (27) found that increased SULF2 expression was positively related to the clinical stage of head and neck squamous carcinoma. Thus, it seemed reasonable for us to suppose an oncogene role for SULF2 in cervical cancer. In addition, p53 activation in liver cancer cells could upregulate the expression of SULF2, while suppression of SULF2 by integrating RNA interference led to the impaired senescence response of cells induced by p53, suggesting that SULF2 might be a key downstream element for p53 function (28). Microarray analysis also revealed that the genetic or pharmacological interference of p53 may directly affect SULF2 expression, and SULF2 was confirmed to be a novel transcription target of p53 (28). Notably, previous evidence has demonstrated a close association of the significant upregulation of p53 with the progression of cervical cancer (29,30). These findings may provide another possibility wherein the increased expression of SULF2 in cervical cancer might be partially induced by p53 upregulation.

Furthermore, the *in vitro* experiment conducted with HeLa cells also indicated that SULF2 overexpression promoted cell proliferation, invasion, and migration while inhibiting apoptosis, which can, however, be reversed by SULF2 inhibition. Coincidently, other reports have also proven the important role of SULF2 in the progression of other tumors, such as lung cancer, breast cancer, and prostate cancer (13,31,32). In further experiments, we treated HeLa cells with LY294002 (an AKT inhibitor) and U0126 (an ERK1/2 inhibitor), and the results showed that both LY294002 and U0126 abolished the promoting effect of SULF2 on the growth of cervical cancer cells. Similarly, Tao et al. (33) also noted that inhibiting SULF2 can suppress the proliferation, cell cycle, and metastasis of HT29 cells (a colorectal cancer cell line) by downregulating p-AKT and p-ERK1/2. SULF2 inhibition could decrease the expression of cyclin D1 and Bcl-2 to promote the apoptosis of liver cancer cells, as reported by Lai et al. (10). The results of this study also demonstrated that SULF2 overexpression can promote the expression of cyclin D1, p-Akt/Akt, p-ERK/ERK, and Bax/Bcl-2, while the opposite alterations were obtained after SULF2 inhibition. According to the work of Jeyamohan et al. (34), inactivation of AKT could upregulate Bax and downregulate Bcl-2, thereby facilitating the apoptosis of cervical cancer cells. Evidence has shown that cyclin D1, as a major regulator in cell proliferation, can modulate the G1/S transition (35). Activated ERK1/2 can induce the expression of cyclin D1 by acting on the FOS family and myc to promote the transition from G1 to S, finally resulting in the malignant proliferation of cells (36). The Bcl-2 family has been shown to play a crucial role in the regulation of cell apoptosis, while the anti-apoptotic role of activated ERK1/2 was mainly achieved by the phosphorylation of Bcl-2, activation of transcription factors, and interference of TRAIL, thereby accelerating the growth of tumor cells (37). Moreover, the activation of ERK can increase the expression of invasion-related genes, including MMP-2 and MMP-9, thereby promoting the invasion and migration of tumor cells (38). Nevertheless, AKT, as a key signaling molecule in the PI3K signaling pathway, was able to be activated by PI3K through phosphorylation to form p-Akt, which has phosphokinase activity and can increase the transcription activity of NF-κB to enhance the proliferation and invasion of tumor cells (39). Meanwhile, p-Akt was reported to inhibit Bax and activate caspase, which can enhance the activity of NF-κB while increasing Bcl-2; as such, the endogenous apoptotic pathway was blocked to abolish the apoptosis of tumor cells (39). Taken together, inhibition of SULF expression can suppress the growth of tumor cells by decreasing the activation of ERK1/2 and AKT to alter the expression of proliferation-related downstream proteins.

Lastly, the xenograft models in nude mice were constructed to validate the regulatory effect of SULF2 on cervical cancer growth *in vivo*. Consequently, SULF2 siRNA inhibited the growth of xenograft tumors, with a significant decrease in tumor weight. Ki67, due to its relatively short half-life and its long-lasting positive expression throughout the whole miotic phase, can efficiently reflect the proliferative activity of cells (40). Thus, the decreased Ki67 expression in the nude mice treated with SULF2 siRNA suggested that SULF2 siRNA limited the proliferation and growth of xenograft tumor cells *in vivo*.

Overall, the present work indicated that SULF2 was upregulated in cervical cancer, while downregulation of SULF2 suppressed the activity of the ERK1/2 and AKT signaling pathways. Furthermore, the proliferation, invasion, and migration of cervical cancer cells were obviously inhibited, with an increase in the apoptotic rate. Hence, SULF2 may be a novel target in the treatment of cervical cancer.
